# The impact of media coverage on the transmission dynamics of human influenza

**DOI:** 10.1186/1471-2458-11-S1-S5

**Published:** 2011-02-25

**Authors:** Jean M Tchuenche, Nothabo Dube, Claver P Bhunu, Robert J Smith?, Chris T Bauch

**Affiliations:** 1 Department of Mathematics and Statistics, University of Guelph, Guelph, Ontario N1G 2W1, Canada; 2 Department of Applied Mathematics, National University of Science and Technology, Box AC 939 Ascot, Bulawayo, Zimbabwe; 3 Department of Mathematics and Faculty of Medicine, The University of Ottawa, 585 King Edward Ave, Ottawa ON K1N 6N5, Canada

## Abstract

**Background:**

There is an urgent need to understand how the provision of information influences individual risk perception and how this in turn shapes the evolution of epidemics. Individuals are influenced by information in complex and unpredictable ways. Emerging infectious diseases, such as the recent swine flu epidemic, may be particular hotspots for a media-fueled rush to vaccination; conversely, seasonal diseases may receive little media attention, despite their high mortality rate, due to their perceived lack of newness.

**Methods:**

We formulate a deterministic transmission and vaccination model to investigate the effects of media coverage on the transmission dynamics of influenza. The population is subdivided into different classes according to their disease status. The compartmental model includes the effect of media coverage on reporting the number of infections as well as the number of individuals successfully vaccinated.

**Results:**

A threshold parameter (the basic reproductive ratio) is analytically derived and used to discuss the local stability of the disease-free steady state. The impact of costs that can be incurred, which include vaccination, education, implementation and campaigns on media coverage, are also investigated using optimal control theory. A simplified version of the model with pulse vaccination shows that the media can trigger a vaccinating panic if the vaccine is imperfect and simplified messages result in the vaccinated mixing with the infectives without regard to disease risk.

**Conclusions:**

The effects of media on an outbreak are complex. Simplified understandings of disease epidemiology, propogated through media soundbites, may make the disease significantly worse.

## Introduction

Infectious diseases are responsible for a quarter of all deaths in the world annually, the vast majority occurring in low- and middle-income countries [1]. There are diseases such as SARS and flu that exhibit some distinct features such as rapid spatial spread and visible symptoms [2]. These features, associated with the increasing trend of globalization and the development of information technology, are expected to be shared by other emerging/re-emerging infectious diseases. It is therefore important to refine classical mathematical models to reflect these features by adding the dimensions of massive news coverage that have great influence not only on the individual behaviours but also on the formation and implementation of public intervention and control policies [2].

People’s response to the threat of disease is dependent on their perception of risk, which is influenced by public and private information disseminated widely by the media. While government agencies for disease control and prevention may attempt to contain the disease [3], the general information disseminated to the public is often restricted to simply reporting the number of infections and deaths. Mass media are widely acknowleged as key tools in risk communication [4,5], but have been criticised for making risk a spectacle to capitalise on audience anxiety [6,7].

The original interpretation of media effects in communication theory was a “hypodermic needle” or “magic bullet” theory of the mass media. Early communication theorists [8,9] imagined that a particular media message would be directly injected into the minds of media spectators. This theory of media effects, in which the mass media has a direct and rapid influence on everyday understanding, has been substantially revised. Contemporary media studies analyses how media consumers might only partially accept a particular media message [10], how the media is shaped by dominant cultural norms [11,12] and how media consumers resist dominant media messages [13,14]. It follows that media effects may sway people into panic (eg swine flu), especially with a disease where scientific evidence is thin or nonexistent. Conversely, media may have little effect on seasonal diseases (eg regular influenza).

Media reporting plays a key role in the perception, management and even creation of crisis [6]. Since media reports are retrievable and because the messages are widely distributed, they gain authority as an intersubjective anchorage for personal recollection [4]. At times of crisis, non-state-controlled media thrive, while state-controlled media are usually rewarded for creating an illusion of normalcy [6]. Media exposure and attention partially mediate the effects of variables such as demographics and personal experience on risk judgments [5]. The role of media coverage on disease outbreaks is thus crucial and should be given prominence in the study of disease dynamics.

Klein *et al*., [15] noted that much more research is needed to understand how provision of information influences individual risk perception and how it shapes the evolution of epidemics; for example, individuals may overprotect, which can have additional consequences for the spread of disease. An example of such complex dynamics is the 1994 outbreak of plague in a state in India: after the announcement of the disease, many people fled the state of Surat in an effort to escape the disease, thus carrying the disease to other parts of the country [16]. Even though information on the number of cases and deaths can have an adverse effect, the number of those vaccinated has not been given prominence.

A handful of mathematical models have described the impact of media coverage on the transmission dynamics of infectious diseases. Liu *et al.*[2] examined the potential for multiple outbreaks and sustained oscillations of emerging infectious diseases due to the psychological impact from reported numbers of infectious and hospitalized individuals. Liu & Cui [3] analysed a compartment model that described the spread and control of an infectious disease under the influence of media coverage. Li & Cui [17] incorporated constant and pulse vaccination in SIS epidemic models with media coverage. Cui *et al*., [18] showed that when the media impact is sufficiently strong, their model – with incidence rate being of the exponential form capturing the alertness to the disease of each susceptible individual in the population – exhibits multiple positive equilibria (also see [2]) which poses a challenge to the prediction and control of the outbreaks of infectious diseases.

The aim of this study is to investigate the impact of media coverage on the spread and control of an influenza strain when a vaccine is available, and where the media reporting of both disease dynamics and vaccination is high. Vaccination is one of the most effective tools for reducing the burden of infectious diseases [19]. However, despite their public-health benefit, vaccination programs face obstacles. Individuals often refuse or avoid vaccinations they perceive to be risky. Recently, rumours that the polio vaccine could cause sterility and spread HIV have hampered polio eradication in Nigeria [20], while misplaced fears of autism in the developed world have stoked vaccination fears [21]. Reporting the number of individuals who vaccinate may have a positive effect on the disease transmission by increasing the vaccination rate.

Conversely, behavioural interventions can also have an enormous effect on the course of a disease [22,23] Our model considers the same contact rate after a media alert, as proposed by Liu & Cui [3], but there are fundamental differences in both models. They consider the classical *SIR* type model, while vaccination is included in ours to reflect transmission dynamics of human influenza.

## Model framework

We divide the population (*N*) into four sub-populations, according to their disease status: susceptible (*S*), vaccinated (*V* ), infected (*I*), and recovered (*R*). Our model monitors the dynamics of influenza based on a single strain without effective cross-immunity against the strain. The susceptible population is increased by recruitment of individuals (either by birth or immigration), and by the loss of immunity, acquired through previous vaccination or natural infection. This population is reduced through vaccination (moving to class *V* ), infection (moving to class *I*) and by natural death or emigration. The population of vaccinated individuals is increased by vaccination of susceptible individuals. Since the vaccine does not confer immunity to all vaccine recipients, vaccinated individuals may become infected, but at a lower rate than unvaccinated. The vaccinated class is thus diminished by this infection (moving to class *I*) by waning of vaccine-based immunity (moving to class *S*) and by natural death. The population of infected individuals is increased by infection of susceptibles, including those who remain susceptible despite being vaccinated. It is diminished by natural death, death due to disease and by recovery from the disease (moving to class *R*). The recovered class is increased by individuals recovering from their infection and is decreased as individuals succumb to natural death. Media coverage is introduced into the model via a saturated incidence function.

**Figure 1:**
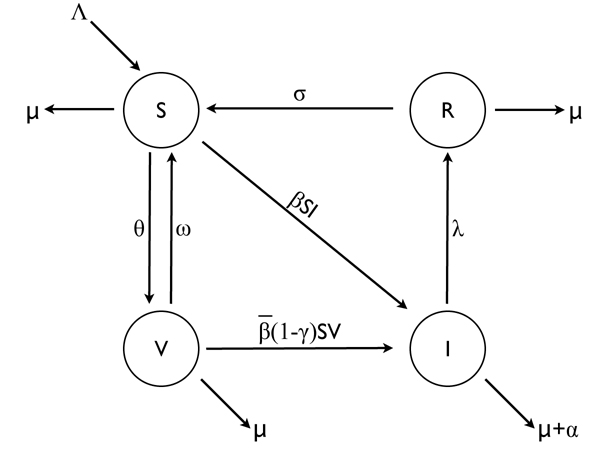
**The model** Schematic model flow diagram

A schematic model flowchart is depicted in Figure 1.

### Model equations

The transmission model with media coverage is given by the following deterministic system of nonlinear ordinary differential equations:(1)(2)(3)(4)

where Λ is the rate at which individuals are recruited into the population (recruitment of infectives is ignored for now); *θ* is the rate at which susceptible individuals receive the vaccine; *µ* is the the rate at which people leave the population, through natural death or emigration. We assume this rate to be the same for all sub-populations. *β*_1_ is the rate at which susceptibles get infected; *ω* is the rate at which vaccine-based immunity wanes; *γ* is the vaccine efficacy; *α* is the death rate due to the infection; and *λ* is the recovery rate from infection. The terms  and  measure the effect of reduction of the contact rate when infectious and vaccinated individuals are reported in the media [2,3,18]. The half-saturation constant *m_I_* > 0 reflects the impact of media coverage on the contact transmission. The function  is a continuous bounded function which takes into account disease saturation or psychological effects [24]. We note that recovered individuals cannot be vaccinated. Also, a vaccinated individual who gets infected and then recovers will return to the susceptible class with no vaccine protection. This is true even if *ω* is quite small but *σ* and *λ* are large. For example, if vaccination lasts three years, but recovery and loss of immunity takes 6 months, then we are assuming this person is subsequently unvaccinated.

In the Michaelis-Menten functional response, the rate at which information is spread by the media rises as infectives increase, but eventually levels off at a plateau (or asymptote) at which the information (rate) remains constant (i.e. it has reached a maximum number of individuals due to information saturation) regardless of the increase in infections. Such dynamics can easily be observed in the spread of rumours, gossip and jokes (also known as randomized broadcast) [25,26]. This constant coverage is extended by examining more complex effects which involve more than just reducing contacts down the line. The news in particular is extremely fickle so that what is news one day may be forgotten about next week; including the media effects in some more sophisticated way such as by an impulsive pulsing is also investigated. The limited power of the infection due to contact is accounted for by the saturation incidence. The first available information is the reported number of infected individuals when the disease is emerging. We assume that media coverage can slow but not prevent disease spread, so *β*_1_ ≥ *β*_2_ and *β*_1_ ≥ *β*_3_.

The above model is closely related to those in [27,28] to analyze the transmission dynamics of human influenza, but there are some differences. In [27], the authors consider the inflow of infective immigrants, while in [28] the model includes treatment. Neither of these are considered here. Our model is clearly a crude reflection of the complicated nonlinear phenomena of the transmission dynamics, and it does not incorporate the self-control property due to the change of avoidance patterns of individuals at different stages of the infectious process [2]. News coverage may have a significant impact on avoidance behaviours at both individual and society levels, which may reduce the effective contact between susceptible and infectious individuals; we include this via a saturation incidence functional response.

Since the model monitors human populations, all the variables and parameters of the model are nonnegative. Based on biological considerations the system of equations (1)-(4) will be studied in the following region,

which is positively invariant and attracting (thus, the model is mathematically and epidemiologically well-posed); it is therefore sufficient to consider solutions in Ω. Existence, uniqueness and continuation results for model system (1)-(4) hold in this region and all solutions of this system starting in Ω remain in Ω for all *t* ≥ 0.

### Stability of the equilibrium states

The disease-free equilibrium of the system is given by

The endemic equilibrium of the system is given by

It satisfies  and

where *h*_1_(*I*) = *m*_1_ + *I*, *h*_2_(*I*) = (*θ* + *µ* + *β*_1_*I*)*h*_1_(*I*) – *β*_2_*I*^2^, *h*_3_(*I*) = (*θ + ω* + (1 – *γ*)*β*_1_*I*)*h*_1_(*I*) – *β*_3_(1 – *γ*)*I*^2^. Substituting the above into the second equation at equilibrium will yield the expression for *Î* after some rearrangement. For illustration, suppose *θ* = *β*_2_ = *β*_3_ = 0. Then the endemic equilibrium satisfies

where Î is the positive solution to the quadratic

The basic reproductive ratio, *R_v_*, is defined as the expected number of secondary infections caused by an infective individual upon entering a totally susceptible population [29-31]. This quantity is not only important in describing the infectious power of the disease, but can also can supply information for controlling the spread of the disease [32]. The linear stability of *E_v0_* is governed by the basic reproductive ratio *R_v_.* Using the next-generation method [31], we have

and

The basic reproductive ratio is the spectral radius *ρ*(*FV*^–1^) which is(5)

### Local stability of the disease-free equilibrium

**Lemma 1***The disease-free equilibrium E_v_*_0 _*is locally asymptotically stable if R_v_* < 1*, and unstable if R_v_* > 1*.*

**Proof.** The Jacobian of the system evaluated at *E_v0_* is given by

The eigenvalues of *J*_*E*_*V*0__ are

For local stability of the disease-free equilibrium, we require that all the eigenvalues be negative. Three of the eigenvalues satisfy this condition while ς_2_ < 0 implies that *R_v_* < 1 and, consequently, all the eigenvalues of the Jacobian matrix above have negative real part. Thus, the disease-free equilibrium is locally asymptotically stable.

### Global stability of the disease-free equilibrium

We adopt the method of Castillo-Chavez *et al,*[33] and we rewrite the set of model equations in the form

with *G*(*X_G_*,0) = 0. *X_G_* ∈ ℝ^3^ denotes the number of uninfected classes and *Z_G_* ∈ ℝ denotes the number of infected classes.  denotes the disease-free equilibrium of the system where  For the set of equations in (1)-(4), we set *X_G_* = (*S*, *V*, *R*) and *Z_G_* = (*I*)*.* The conditions (H1) and (H2) below must be met for global stability.

(H1) For  is globally asymptotically stable.

(H2) *G*(*X_G_*, *Z_G_*) = *A_G_Z_G_* – *Ĝ*(*X_G_*, *Z_G_*), *Ĝ*(*X_G_*, *Z_G_*) ≥ 0 for (*X_G_*, *Z_G_*) ∈ Ω where  is an *M*-matrix (the off-diagonal elements of A are nonnegative) and Ω is the region where the model makes biological sense.

If the above two conditions are satisfied, then the following theorem holds.

**Theorem 2 ***(Castillo-Chavez et al,*[33]*): The fixed point**is a globally stable equilibrium of (2.28) provided that R_v_* < 1 *and that assumptions (H1) and (H2) are satisfied.*

Therefore, *E_v_*_0_ is globally asymptotically stable (GAS) since *Ĝ*(*X_G_,Z_G_*) > 0. The GAS of *E_v_*_0_ excludes any possibility of the phenomenon of backward bifurcation. We note that the GAS of the DFE *E_v_*_0_ when *σ* = 0 is straightforward.

## The optimal control model

Our objective in this section is to extend the initial model to include two intervention methods, called controls, represented as functions of time and assigned reasonable upper and lower bounds, each representing a possible method of influenza intervention. Using optimal control theory and numerical simulations, we determine the benefit of vaccination and media coverage when the latter has positive or negative effect on the former.

We will integrate the essential components into one SIVR-type model to accommodate the dynamics of an influenza outbreak determined by population-specific parameters such as the effect of contact reduction when infectious and vaccinated individuals are reported in the media.

Let *u_v_* and *u_m_* be the control variables for vaccination and media coverage respectively. Thus, model (1)-(4) now reads(6)(7)(8)(9)

A balance of multiple intervention methods can differ between populations. A successful mitigation scheme is one which reduces influenza-related deaths with minimal cost. A control scheme is assumed to be optimal if it maximizes the objective functional(10)

The first two terms represent the benefit of the susceptible and vaccinated populations. The parameters *B*_1_ and *B*_2_ represent the weight constraints for the infected population and the control, respectively. They can also represent balancing coefficients transforming the integral into dollars expended over a finite time period of *T* days [34]. The goal is to maximize the populations of susceptible and vaccinated individuals, minimize the population of infectives, maximize the benefits of media coverage and vaccination, while minimizing the systemic costs of both media coverage and vaccination. The value *u_v_*(*t*) = *u_m_*(*t*) = 1 represents the maximal control due to vaccination and media coverage, respectively. The terms  and  represent the maximal cost of education, implementation and campaigns on both vaccination and media coverage. *S*(*t*) and *V*(*t*) account for the fitness of the susceptible and the vaccinated groups as a result of a reduction in the rate at which the vaccine wanes, and vaccination and treatment efforts are implemented [35]. We thus seek optimal controls  and  such that

where *U* = {(*u_v_*, *u_m_*)|*u_v_*, *u_m_* measurable, 0 ≤ *a*_11_ ≤ *u_v_* ≤ *b*_11_ ≤ 1, 0 ≤ *a*_22_ ≤ *u_m_* ≤ *b*_22_ ≤ 1, *t* ∈ [0, *t_f_*]} is the control set, with *t* ∈ [*t*_0_, *t_f_*]. The basic framework of this problem is to characterize the optimal control.

### Existence of an optimal control

The existence of an optimal control can be obtained by using a result by Joshi [36] and Fister *et al.*[37].

**Theorem 3***Consider the control problem with the system of Equations (4.1)-(4.4). There exists an optimal control**such that max*

**Proof.** To prove this theorem on the existence of an optimal control, we use a result from Fleming and Rishel [38] (Theorem 4.1 pp. 68-69), where the following properties must be satisfied.

1. The set of controls and corresponding state variables is nonempty.

2. The control set *U* is closed and convex.

3. The right-hand side of the state system is bounded above by a linear function in the state and control.

4. The integrand of the functional is concave on *U* and is bounded above by *c*_2_ – *c*_1_(*|u_v_|^k^* + *|u_m_|^k^*), where *c*_1_, *c*_2_ > 0 and *k* > 1.

An existence result in Lukes [39] (Theorem 9.2.1) for the system of equations (6)-(9) for bounded coefficients is used to give the first condition. The control set is closed and convex by definition. The right-hand side of the state system (Equations (4.1)-(4.4)) satisfies Condition 3 since the state solutions are a priori bounded. The integrand in the objective functional, , is concave on *U*. Furthermore, *c*_1_, *c*_2_ > 0 and *k* > 1, so(11)

Therefore, the optimal control exists, since the left-hand side of (11) is bounded; consequently, the states are bounded.

Since there exists an optimal control for maximizing the functional (10) subject to equations (6)-(9), we use Pontryagin’s Maximum Principle to derive the necessary conditions for this optimal control. Pontryagin’s Maximum Principle introduces adjoint functions that allow us to attach our state system (of differential equations), to our objective functional. After first showing existence of optimal controls, this principle can be used to obtain the differential equations for the adjoint variables, corresponding boundary conditions and the characterization of an optimal control  and . This characterization gives a representation of an optimal control in terms of the state and adjoint functions. Also, this principle converts the problem of minimizing the objective functional subject to the state system into minimizing either the Lagrangian or the Hamiltonian with respect to the controls (bounded measurable functions) at each time *t*[40]. The Lagrangian is defined as

where *w*_11_(*t*) ≥ 0, *w*_12_(*t*) ≥ 0 are penalty multipliers satisfying *w*_11_(*t*)(*a*_11_ – *u_v_*(*t*)) + *w*_12_(*t*)(*u_v_*(*t*) – *b*_11_) at the optimal , and *w*_21_(*t*) ≥ 0, *w*_22_(*t*) ≥ 0 are penalty multipliers satisfying *w*_21_(*t*)(*a*_22_ – *u_m_*(*t*)) + *w*_22_(*t*)(*u_m_*(*t*) – *b*_22_) at the optimal .

Given optimal controls  and  , and solutions of the corresponding state system (6)-(9), there exist adjoint variables λ_i_, for i = 1, 2, 3, 4 satisfying the following equations


with transversality conditions *λ_i_*[*t_f_*] = 0, for *i* = 1*,* 2*,* 3*,* 4. To determine the interior maximum of our Lagrangian, we take the partial derivatives of L with respect to *u_v_* and *u_m_*, respectively, and set it to zero. Thus,

To determine an explicit expression for our controls ,  (without *w*_11_*, w*_12_, *w*_21_, *w*_22_), a standard optimality technique is utilized. The following cases are considered to determine a specific characterization of the optimal control.

**Case 1:** Optimality of 

1. On the set . Hence, the optimal control is

2. On the set .We have

or

since *w*_12_ ≥ 0.

3. On the set . Hence

or

Combining all the three sub-cases in a compact form gives(12)

**Case 2:** Optimality of 

1. On the set . We have

2. On the set . We have

or

since *w*_22_ ≥ 0.

3. On the set . Hence

or

Combining all the three sub-cases in a compact form gives(13)

### The optimal system

The optimality system consists of the state system coupled with the adjoint system, with the initial conditions, the transversality conditions and the characterization of the optimal control:

where  and  are given by expressions (12) and (13), respectively, with *S*(0) = *S*_0_, *I*(0) = *I*_0_, *V*(0) = *V*_0_, *R*(0) = *R*_0_ and *λ_i_*[*t_f_*] = 0 for *i* = 1*,··· ,*4. Due to the a priori boundedness of the state and adjoint functions and the resulting Lipschitz structure of the ODEs, we obtain the uniqueness of the optimal control for small [*t_f_*] [36]. The uniqueness of the optimal control follows from the uniqueness of the optimality system.

**Figure 2:**
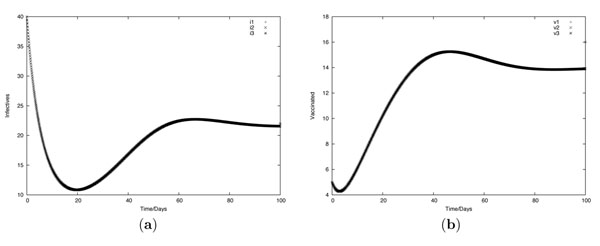
**Optimality effect when the weight constraint for the infected population varies and media has a beneficial effect on the vaccine.** Graphs of the optimality system when media coverage has a beneficial effect on the vaccination rate and when the weight constraint for the infected population varies. (a) Infected individuals. (b) Vaccinated individuals. Initial conditions: *S*(0) = 20*.*0, *I*(0) = 25*.*0, *V*(0) = 50*.*0, *R*(0) = 40*.*0*.* The value of the weights used are (i) *B*1 = 0*.*0025 corresponds to variables with subscript 1 (++), (ii) *B*1 = 25*.*0 corresponds to variables with subscript 2 (xx), (iii) *B*1 = 250000*.*0 corresponds to variables with subscript 3 (**). The value *B*2 = 0*.*0025 is kept constant in all three cases.

**Figure 3:**
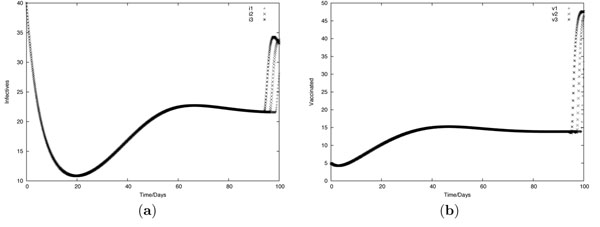
**Optimality effect when the weight constraint for the control varies and media has a beneficial effect on the vaccine.** Graphs of the optimality system when media coverage has a beneficial effect on the vaccination rate and when the weight constraint for the control varies. (a) Graph of infectives, (b) Graph of vaccinated individuals. Initial conditions: *S*(0) = 20*.*0, *I*(0) = 25*.*0, *V*(0) = 50*.*0, *R*(0) = 40*.*0. The value of the weights used are (i) *B*2 = 25*.*0 corresponds to variables with subscript 1 (++), (ii) *B*2 = 2500*.*0 corresponds to variables with subscript 2 (xx), (iii) *B*2 = 250000*.*0 corresponds to variables with subscript 3 (**). The value *B*1 = 0*.*0025 is kept constant in all three cases.

**Figure 4:**
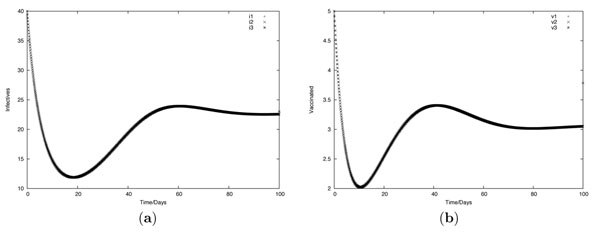
**Optimality effect when the weight constraint for the infected population varies and media has an adverse effect on the vaccine.** Graphical representation of the optimality system when media coverage has an adverse effect on the vaccination rate and when the weight constraint for the infected population varies. (a) Graph of infectives. (b) Graph of vaccinated individuals. Initial conditions: *S*(0) = 20*.*0, *I*(0) = 25*.*0, *V*(0) = 50*.*0, *R*(0) = 40*.*0. The value of the weights used are (i) *B*1 = 0*.*0025 corresponds to variables with subscript 1 (++), (ii) *B*1 = 25*.*0 corresponds to variables with subscript 2 (xx), (iii) *B*1 = 250000*.*0 corresponds to variables with subscript 3 (**). The value *B*2 = 0*.*0025 is kept constant in all three cases.

**Figure 5:**
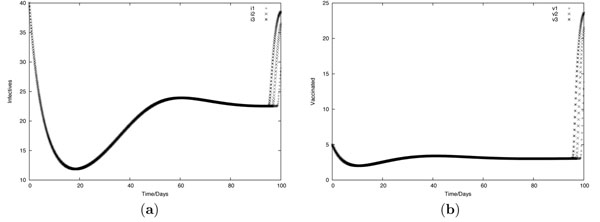
**Optimality effect when the weight constraint for the control varies and media has an adverse effect on the vaccine.** Graphs of the optimality system when media coverage has an adverse effect on the vaccination rate and when the weight constraint for the control population varies. (a) Graph of infectives. (b) Graph of vaccinated individuals. Initial conditions: *S*(0) = 20*.*0, *I*(0) = 25*.*0, *V*(0) = 50*.*0, *R*(0) = 40*.*0. The value of the weights used are (i) *B*2 = 25*.*0 corresponds to variables with subscript 1 (++), (ii) *B*2 = 2500*.*0 corresponds to variables with subscript 2 (xx), (iii) *B*2 = 250000*.*0 corresponds to variables with subscript 3 (**). The value *B*1 = 0*.*0025 is kept constant in all three cases.

The state system of differential equations and the adjoint system of differential equations together with the control characterization above form the optimality system solved numerically and depicted in Figures 2, 3, 4, 5.

### The model with pulse vaccination

The general model with pulse vaccination is given as

for *t* ≠ *t_k_*, where *t_k_* is the time of the *k*th vaccination. We may have *t*_*k*+1_ – *t_k_* either constant or not, as we choose. The impulsive effect is given by

Δ*S* = –*θS*

Δ*V* = *θS*

when *t* = *t_k_.* Here,  is the change in state at the impulse time.

In this model, vaccination occurs at fixed times, not continuously. This is closer to reality, since vaccination centres are only open at certain times, when people may get vaccinated in waves. Similarly, media stories tend to clump together, so that a big news story occurs on one day, which may trigger a short period of intense vaccination. We shall use a simplified version of this model to illustrate the possibility that media may have an adverse effect.

### Adverse effects

Consider the following scenario. At the onset of the outbreak, the media - and hence the general population - is unaware of the disease and thus nobody gets the vaccine, allowing the disease to spread in its initial stages. At some point, there is a critical number of infected individuals, whereupon people are sufficiently aware of the infection to change their behaviour. We suppose that, initially, new infected people arrive at fixed times.

We further assume that vaccinated people mix more than susceptibles. In this case, people who are vaccinated feel confident enough to mix with the infected, even though they may still have the possibility to contract the virus. This might be the case for health-care workers, for instance, who get vaccinated and then have to tend to the sick.

Mathematically, we have a threshold for the critical number of infectives, *I*_crit_*.*

For *I* <*I*_crit_, this model would look like

For *I* >*I*_crit_, the model becomes

with *β*_4_ – *β*_6_ ≥ 0.

However, to illustrate the adverse affect, we shall simplify the model even further. For a short timescale, we can assume recovery is permanent, so *σ* = 0. Thus, we can ignore the *R* equation.

For *I* <*I*_crit_, we assume that there is no mixing, but rather that new infectives arrive impulsively into the system at fixed times *t_k_* and in numbers *I^i^,* where *I^i^* ≪ *I*_crit_. (If the new infectives arrive at irregular times, then the broad results will be unchanged.)

For *I* >*I*_crit_, fear of the disease keeps susceptibles from mixing with the infected, but the vaccinated will.

Thus *β*_4_ = *β*_6_ = 0. Since *I^i^* ≪ *I*_crit_, we can assume that, for *I* >*I*_crit_, the effects of new infectives are negligible.

The model then becomes(14)(15)(16)(17)

for *I* <*I*_crit_ and(18)(19)(20)

for *I* >*I*_crit_.

Thus, the effects of the media are to trigger a vaccinating panic whenever the number of infectives is large enough. We kept the model with impulse vaccination as simple as possible since even this simplified version shows that media reports could have an adverse effect.

Suppose new infectives appear regularly, so that *t_k_*_+1_ – *t_k_* = *τ*. (If not, the analysis generalizes quite easily.) For *t_k_* <*t* <*t_k_*_+1_, we have

where  is the value immediately after the *k*th impulse. Then, since the period is constant, we have

This is a recursion relation with solution

Consequently,

Thus, if *m*^+^ >*I*_crit_, then eventually the system will switch from model (14)-(17) to model (18)-(20). The endemic equilibrium in model (18)-(20) satisfies

The Jacobian is

At the endemic equilibrium, . Thus, we have

The characteristic equation is

It follows that the endemic equilibrium is stable if *Î* >*I*_crit_. Thus, even in an extremely simplified version of the model, the media may make things significantly worse than if no media effect were included. We kept this model deliberately simple, partly for mathematical tractability and partly to show that the media effects apply even in this idealised scenario.

Note that, in reality, the fluctuations would apply in the upper region as well, making the actual value even

larger. In the lower region, we ignored interaction between susceptibles and infectives (ie we assume *β*_4_ = *β*_6_ = 0). The effect of including these terms would be to slow the exponential decay between impulses (or possibly cause it to increase). This would only increase the effect seen here.

In summary, a small series of outbreaks that would equilibrate at some maximum level *m*^+^ >*I*_crit_ will, as a result of the media, instead equilibrate at a much larger value *I* >*m*^+^ >*I*_crit_. The driving factor here is if an imperfect vaccine causes overconfidence, so that people who have been vaccinated mix significantly more with infectives than susceptibles do. If this happens (as would be quite likely; most people who have been vaccinated feel invulnerable, even if the vaccine is not perfect, largely thanks to media oversimplifications), then the media effect is likely to be adverse. A simplified version of the model with pulse vaccination shows that the media can make things worse, if the vaccine is imperfect because the vaccinated mix over-confidently with the infectives.

## Numerical simulations

We now return to model (6)-(9) and illustrate some of the properties discussed in the previous sections. The parameter values that we use for numerical simulations are in Table 1. Initial conditions: *S*(0) = 200*.*0, *I*(0) = 1*.*0, *V*(0) = 10*.*0, *R*(0) = 0*.*0*.* The parameter *θ* varies between 0*.*3 and 0*.*7 with an average of 0*.*5 [41]. We consider an imperfect flu vaccine for which the waning rate is about 0*.*15. The relationship between *β*_2_ and *β*_3_ is not very obvious; consequently, we can either assume equality or that the former is slightly greater than the latter. Transmission dynamics of infectious diseases with and without media coverage have already been carried out in previous studies, but these models do not account for the vaccination coverage. Therefore, we illustrate some numerical results for the model with optimal control when media coverage has (i) a beneficial effect (see Figures 2 and 3) (ii) and adverse effect on the vaccination rate (see Figures 4 and 5).

**Table 1:** Parameter values

Parameter	Symbol	Value	Units	Reference
Recruitment rate	Λ	5.0	People day^–1^	[3]
Rate at which vaccine wanes	*ω*	0.15	day^–1^	Assumed
Vaccine uptake rate	*θ*	0.3-0.7	day^–1^	[41]
Natural death rate	*µ*	0.02	day^–1^	[3]
Infection rate	*β*_1_	0.02	people^–1^day^–1^	[3]
Loss of immunity	*σ*	0.01	day^–1^	[3]
Vaccine efficacy	*γ*	0.8	(unitless)	[3]
Infection death rate	*α*	0.1	day^–1^	[3]
Recovery rate of Infectives	*λ*	0.05	day^–1^	[3]
Reaction due to media coverage	*m_I_*	10.0	people	Assumed

Media coverage model parameters, their interpretations and values

The optimality system is solved using an iterative method with a fourth order Runge-Kutta scheme. Starting with a guess for the adjoint variables, the state equations are solved forward in time. Then the state values obtained are used to solve the adjoint equations backward in time; the iterations continue until convergence. Simulations are carried out to determine how maximizing media coverage enhances vaccination. The effects of costs that can be incurred, which include education, implementation and campaigns on media coverage, are also studied to evaluate how these costs can affect the transmission of human influenza. We increase the value of *B*2 (the cost weight) in Figure 2 to assess how the populations of susceptibles, infectives, vaccinated and recovered individuals are altered. In Figure 3, we investigate how increasing minimization of infectives through increasing the weight *B*1 affects the control of human influenza transmission. We do the same in Figures 4 and 5, respectively, to see how, if media coverage has an adverse effect, the various populations behave. In Figure 4, we vary the cost weight, while in Figure 5, we vary the weight of minimizing infectives.

We note from Figure 2 that, during the initial days, there is a very sharp drop in the population of infected individuals, while other populations show increases. Increasing minimization of infectives, while keeping costs low, can lead to the disease being controllable. The slight rise and fall, after the initial 20 days, in the population of infectives could be attributed to complacency on the part of some individuals (or may be due to oscillations in the system independent of external factors). We find that people tend to relax after the initial shock of the disease threat. However, we note that this is not for long, and this could be attributed to the fact that vaccination levels continue to rise, so as people continue to receive vaccination, infection is controlled. Thus, if costs are kept minimal, and more people are able to access media and vaccination, then infection can be controlled. Both vaccination and media coverage continue at optimum levels as a result of the low costs and minimization of infectives.

From Figure 3, as costs are increased, few people have access to media and vaccination; as a result, low numbers get vaccinated against the disease. In the long run, the infection levels rise. The degree of media coverage and vaccination also decrease as a result of the exorbitant costs. With the little available media coverage and the few vaccinated individuals, we find that, due to information filtration, there is a jump in the vaccination levels, though these only last briefly; as the degree of media coverage and vaccination decrease, so do the vaccination levels.

From Figure 4, even though costs are kept at minimal levels, the negative reports concerning vaccination result in a drastic reduction in the vaccination levels. After some time, we note a slight increase in the vaccination levels; however, these numbers remain very low. This could be due to the fact that, as infection rises, a few will risk getting vaccinated in the hope of being cured. Thus, media coverage can have adverse effects if people’s perception towards the vaccine is negatively influenced by the media.

**Figure 6:**
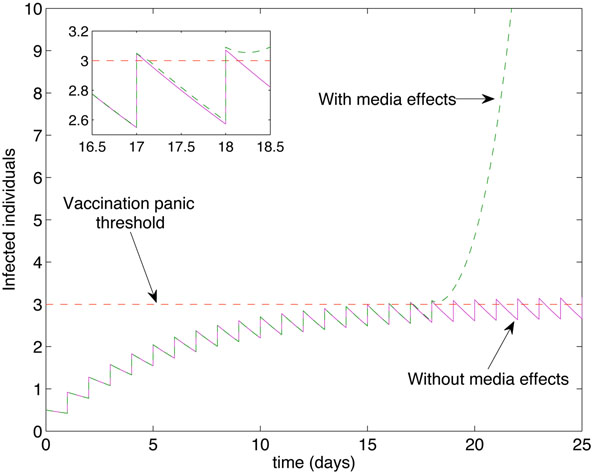
**The vaccination panic threshold** The effect of the vaccination panic threshold using the simplified model (14)-(20). Without media triggering a vaccinating panic, the number of infected individuals remains low (solid purple curve). However, if the media triggers a vaccinating panic, then the number of infected individuals rises sharply (dashed green curve). Inset: Comparison of the two outcomes around the vaccination threshold.

**Figure 7:**
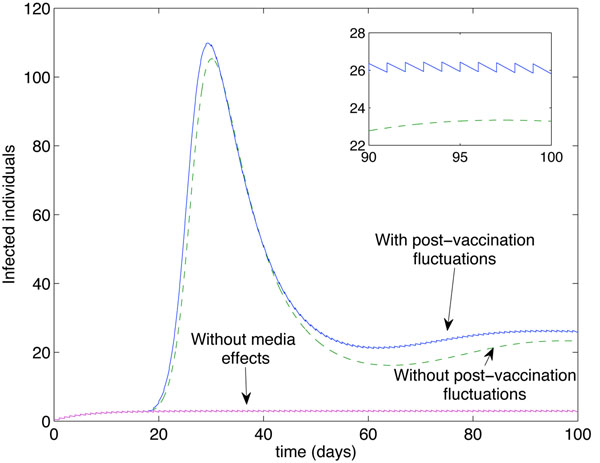
**A media-induced epidemic** Long-term dynamics for the simplified model (15)-(20). Without media effects, the maximum number of infected individuals remains low (solid purple curve). When media effects trigger a vaccinating panic where partially protected vaccinated individuals mix with infectives significantly more than susceptible individuals, a significant outbreak may occur, with the final number of infected individuals much higher than if no media effects had been included (dashed green curve, assuming no fluctuations after the vaccinating panic occurs). The result of including fluctuations above the vaccination panic threshold are also shown (solid blue curve). Inset: magnified view of the fluctuation versus nonfluctuation cases.

In Figure 5, both media coverage and vaccination are eventually withdrawn. Very low numbers get vaccinated. It is only when infection escalates that vaccination levels also increase as some might find it better to try to prevent the infection, despite the negativity towards vaccination in the media. Figure 6 illustrates other potential adverse effects that media may have, if the effect is to trigger a vaccinating panic where vaccinated individuals are not fully protected and mix with infected individuals but susceptible individuals do not. In this case, the number of infected individuals may increase sharply as a result of the media. Figure 7 illustrates the long-term results of a media-induced vaccination panic. Without media effects, the result is a low-level infection. When the media triggers a vaccinating panic, there is a large outbreak, followed by an endemic level of infected individuals significantly higher than the level of infected individuals without the media effects. Note that these examples assume no post-vaccination mixing of susceptible and infected individuals.

**Figure 8:**
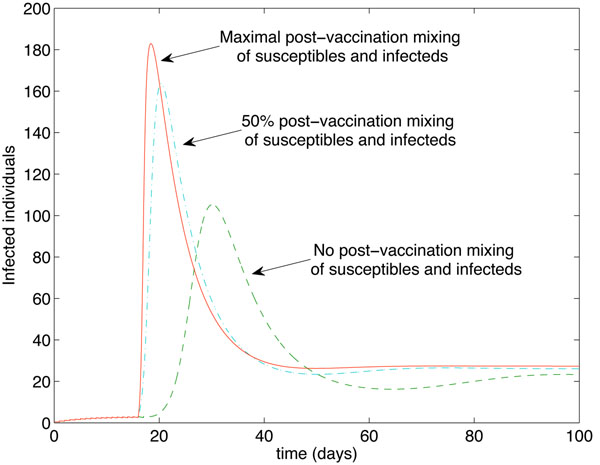
**The effect of different post-panic mixing rates** Comparison of mixing rates between susceptibles and infected individuals after a vaccinating panic. The more susceptibles mix with infecteds, the earlier the outbreak occurs and the larger the number of infected individuals.

Figure 8 illustrates the cases when post-vaccination mixing of susceptible and infected individuals is maximal (*β*_4_ = *β*_5_, *β*_6_ = 0), 50% (*β*_4_ = *β*_5_, *β*_6_ = *β*_4_*/*2) or zero (*β*_4_ = *β*_6_ = 0). Thus, if susceptible and infected individuals mix after a vaccinating panic has occurred, the effect is an earlier outbreak and a larger number of infected individuals.

## Conclusion

Media simplifications can lead to overconfidence in the idea of a vaccine as a cure-all. The result is not just a vaccinating panic and a blow-out epidemic, but a net increase in the endemic equilibrium. Thus, media coverage of an emerging epidemic can fan the flames of fear and also implicitly reinforce an imperfect solution as the only answer.

We have formulated and investigated a simple deterministic vaccination model describing the effects of media coverage on the transmission dynamics of influenza. The media effect due to reporting the number of infections as well as the number of individuals successfully vaccinated is introduced into the compartmental model via a saturated incidence-type function. The impact of costs that can be incurred, which include vaccination, education, implementation and campaigns on media coverage, are also investigated using optimal control theory applied via the Pontryagin’s maximum principle. A simplified version of the model with pulse vaccination shows that the media can have an adverse effect if the vaccine is imperfect and the vaccinated mix over-confidently with the infectives. Numerical simulations are carried out to support the analytical results.

We note, however, that our caricature model is not complete; a more comprehensive study will require interdisciplinary research across traditional boundaries of social, natural, medical sciences and mathematics [2]. Nevertheless, our work provides some insights into the effects of media reporting on the transmission dynamics of infectious diseases for which a vaccine exists. The present study is in no way exhaustive and can be extended in various ways: for example, to investigate the case in which there is media coverage but people ignore it (in which case the vaccination rate is unchanged despite the control). Thus, the effects of media on an outbreak of influenza with a partially effective vaccine may be complicated. While the media may encourage more people to get vaccinated, they may also trigger a vaccinating panic or promote overconfidence in the ability of a vaccine to fully protect against the disease. This may have potentially disastrous consequences in the face of a new pandemic.

## Competing interests

The authors declare that they have no competing interests.

## Authors contributions

JMT, RJS and CTB developed the model. JMT and CTB designed and formulated the study framework and analyzed the model. CPB and ND carried out the optimal control analysis and numerical simulations for Figures 2, 3, 4, 5. RJS wrote the section on adverse effects, some of the introduction, performed numerical simulations for Figures 1,6,7,8, and edited the manuscript. All authors read and approved the final manuscript.
